# The Association of Energy Poverty with Health and Wellbeing in Children in a Mediterranean City

**DOI:** 10.3390/ijerph18115961

**Published:** 2021-06-02

**Authors:** Laura Oliveras, Carme Borrell, Irene González-Pijuan, Mercè Gotsens, María José López, Laia Palència, Lucía Artazcoz, Marc Marí-Dell’Olmo

**Affiliations:** 1 Agència de Salut Pública de Barcelona, 08023 Barcelona, Spain; cborrell@aspb.cat (C.B.); mgotsens@aspb.cat (M.G.); mjlopez@aspb.cat (M.J.L.); lpalenci@aspb.cat (L.P.); lartazco@aspb.cat (L.A.); mmari@aspb.cat (M.M.-D.); 2 Institut d’Investigació Biomèdica Sant Pau (IIB Sant Pau), 08041 Barcelona, Spain; 3 Department of Experimental and Health Sciences, Universitat Pompeu Fabra, 08005 Barcelona, Spain; 4 CIBER Epidemiología y Salud Pública (CIBERESP), 28029 Madrid, Spain; 5 Centre for Regional Economic Social Research, Sheffield Hallam University, Sheffield S1 2LX, UK; irene.gonzalez@esf-cat.org

**Keywords:** energy poverty, fuel poverty, health, health inequalities, social determinants of health, children, southern Europe, urban

## Abstract

Children have been identified as being particularly vulnerable to energy poverty (EP), but little empirical research has addressed the effect of EP on children’s health and wellbeing, especially in southern Europe. In this work we aimed to provide an in-depth description of the distribution of EP by sociodemographic, socioeconomic and housing characteristics, as well as to analyse the association between EP and health and wellbeing in children in Barcelona. We performed a cross-sectional study using data from the Barcelona Health Survey for 2016 (*n* = 481 children under 15 years). We analysed the association between EP and health outcomes through prevalence differences and prevalence ratios (PR) and their 95% confidence interval (CI), using Poisson regression models with robust variance. In Barcelona, 10.6% of children were living in EP and large inequalities were found by sociodemographic, socioeconomic and housing characteristics. EP was strongly associated with poor health in children (PR (95% CI): 7.70 (2.86, 20.72)). Living in EP was also associated with poor mental health (PR (95% CI): 2.46 (1.21, 4.99)) and with more cases of asthma (PR (95% CI): 4.19 (1.47, 11.90)) and overweight (PR (95% CI): 1.50 (1.05, 2.15)) in children. It is urgent to develop specific measures to avoid such serious and unfair health effects on children.

## 1. Introduction

Housing is a key social determinant of health [1]. Children are especially vulnerable to the effects of inadequate housing, because they spend more time at home and because a safe and adequate home is essential for their favourable development and physical and emotional wellbeing [2]. Adequate housing is not just four walls. It involves, among many other factors, basic supplies to carry out reproductive and caregiving activities, such as cooking or cleaning, and to achieve thermal comfort and the use of appliances and other electronic devices enabling effective participation in society [3]. If a household cannot secure a materially and socially required level of energy services, there is a situation of energy poverty (EP) [4].

EP is a major public health problem in the European Union (EU), affecting 9% of the population in 2016 [5]. EP is gaining increasing academic and policy attention in southern European countries because its rates are above the EU average (32.6% in Greece, 24.8% in Portugal or 13.4% in Spain in 2016) [5] and because of the emerging need for space cooling to adapt to climate change and increasingly high temperatures [6]. In Barcelona, for example, 12.4% of the population cannot afford to maintain their dwellings at an adequate temperature during the cold and/or warm months. This average value, however, hides much higher percentages in specific social groups, such as women born in low- and middle-income countries, where the percentage of EP is as high as 24% [7]. Like other social determinants of health, EP follows the classic lines of social stratification and is rarely an isolated problem. It tends to coexist with financial difficulties, unemployment and other material hardship [8,9]. Therefore, specific sociodemographic, socioeconomic and housing characteristics may be related to a disproportionate burden of EP [10]. For example, higher EP rates have been found in single-parent families, families living in market-price rented accommodation and those with food or housing insecurity [8,10,11,12].

Several studies have shown that EP negatively affects people’s health and wellbeing [5,13,14,15,16,17]. Although children have been identified as a particularly vulnerable population group, little empirical research has addressed how, specifically, they are affected by EP, especially in southern Europe. According to the scarce existing literature, the main negative effects of EP on infants and young children concern physical health. In this age range, some studies suggest that EP is associated with a higher risk of health problems, mainly respiratory problems, greater health services use and worse disease course, with more recurrences [18,19,20]. Children living in EP are also at higher risk of under-nutrition or being overweight due to poor nutrition resulting from austerity in expenditure control, purchase of obesogenic products that are usually cheaper or, in more extreme cases, the inability to cook or preserve food [21,22]. Moreover, irregular connection of supplies or the use of alternative heating sources may increase the risk of domestic accidents such as burns or carbon monoxide inhalation [23,24]. EP and its effects on physical health can in turn affect children’s wellbeing and school performance [2,25]. In contrast, among adolescents, EP appears to primarily affect mental health. Difficulties in finding the needed privacy and personal space at home, feeling unhappy with their families and poorly cared for, being afraid of bullying and spending more time in public spaces such as parks or shopping precincts are some of the described effects of EP that can affect the mental health of adolescents and can also increase risk behaviours (e.g., early alcohol and tobacco abuse) [13,15,26]. Recently, a study in China showed that EP had a negative impact on subjective well-being in adolescents aged 10–15 years and suggested that academic performance might be one of the most important mediating mechanisms [27]. Finally, families’ difficulties in paying their utility bills and the accumulation of debt can also affect the mental health of children and adolescents through the influence of economic stress on parental mental health [28], couple interaction and parenting [29].

This study was motivated by the lack of empirical data on how EP affects families differently and its effects on the health and wellbeing of children in southern Europe. Therefore, the aim of this study was to provide an in-depth description of the distribution of EP by sociodemographic, socioeconomic and housing characteristics, as well as to analyse the association between EP and health and wellbeing in children in Barcelona in 2016.

## 2. Materials and Methods

### 2.1. Design, Information Source and Study Population

We performed a cross-sectional study using data from the Barcelona Health Survey for 2016. This survey covers a representative sample of the non-institutionalised population in the city and includes a specific questionnaire for people under the age of 15 years. This under-15 s population includes infants through adolescents, and will henceforth be referred to as children. This study includes all the children who participated in the survey, which represents a sample size of 481 children, sufficient to obtain representative population estimates of percentages with a precision of +/− 4.5% [30]. The questionnaire was completed by the child’s usual caregiver.

### 2.2. Study Variables

#### 2.2.1. Health and Wellbeing 

To assess general health, we used caregiver-reported child health and we dichotomised it into two categories: good health (excellent, very good or good) and poor health (fair or poor) [31]. Mental health was measured using the Strengths and Difficulties Questionnaire (SDQ) for ages 4–17 years. We analysed the total difficulties score, as well as the individual scales of conduct problems, hyperactivity, emotional problems and peer problems. We dichotomised the five scales using the borderline cut-points [32]. We also studied health-related quality of life (HRQoL) for ages 6–14 years through the Kidscreen-10. This is a 10-item questionnaire that provides a Rashed-scaled single score where higher scores indicate better HRQoL [33,34]. Finally, we also analysed two health outcomes that, according to the literature, can be significantly affected by EP, namely asthma and overweight [19,35]. Asthma was assessed through a chronic morbidity checklist, where the caregivers were asked, among others things, if the children suffered from asthma. Overweight was calculated from the height and weight provided by the cargiver. Overweight was defined as a BMI-for-age greater than 1 standard deviation above the WHO Growth Reference median [36,37].

#### 2.2.2. Energy Poverty 

The main EP variable used in this study was constructed from the following two questions: Can the household afford to keep the home at an adequate temperature during the cold months? Can the household afford to keep the home at an adequate temperature during the warm months? EP was considered when the caregiver reported that the household could not afford to keep the home at an adequate temperature during the cold and/or warm months. However, to assess the overlap between different dimensions of EP, the socioeconomic and household conditions variables (described below) also included other primary and secondary EP indicators [38].

#### 2.2.3. Sociodemographics 

We included sex, age, the parents’ country of birth and household composition and we categorize the variables as defined hereafter. The parents’ country of birth was classified into high-income (HI) and low- and middle-income (LMI) countries according to the 2018 World Bank classification [39]. We then classified children into three groups: both parents from HI countries; one from HI and the other from LMI countries; and both from LMI countries. Households were categorised into four groups, single-parent, extended single-parent when the parent and children lived with more people, two-parent and extended two-parent households.

#### 2.2.4. Socioeconomics

We included social class, the ability of the household to afford an unforeseen expenditure of €750 with their own resources (based on one of the primary indicators to measure material deprivation used by the European Commission (European Statistics on Income and Living Conditions [40,41]), arrears on utility bills in the last 12 months (primary EP indicator), food insecurity and housing tenure. We categorize the variables as defined hereafter. Children were assigned the most privileged social class among the parents, based on their current or last occupation. Social class was dichotomised into non-manual and manual class [42]. To measure food insecurity, we used an adaptation of the 6-item US Household Food Security Survey Module, which has been validated for the Hispanic population [43]. Housing tenure was categorised into five categories: paid property, property paying mortgage, rent at market price, social renting and other (re-renting of part of the dwelling, squatting and cession by family members, social services or non-governmental organisations).

#### 2.2.5. Housing Conditions Related to EP

We included three dichotomous variables, which are also secondary EP indicators: dwelling with a leaking roof, damp walls, floors or foundations, or rot in window frames or floors; dwelling without means of heating or with central heating or room-heating appliances, but not used when necessary; and dwelling without an air conditioner or with an air conditioner, but not used when necessary.

For more information on the variables used, as well as to check the specific questions, please consult the Barcelona Health Survey manual, which also includes the complete questionnaire [30].

### 2.3. Data Analysis

First, we categorized the different sociodemographic, socioeconomic and housing conditions variables as detailed in the previous section and we described the sample according to theses variables. Then, we calculated the percentage of EP and its 95% confidence interval (CI) for the total population, as well as by sociodemographic, socioeconomic and housing condition variables and we used the Pearson chi-square test with the second-order Rao-Scott correction to assess differences in EP percentage within categories of each variable [44]. Subsequently, we obtained the prevalence of health indicators among children with and without EP. Finally, we used Poisson regression models with robust variance to assess absolute and relative differences of the prevalence of health indicators among children with and without EP [45]. To do this, we calculated prevalence differences (PD) and prevalence ratios (PR) and their 95%CI, respectively. In the case of the Kidscreen, the only continuous health outcome, we calculated means and their 95%CI for both groups and their association with EP was studied using linear models. EP often occurs in a wider context of material hardship and disadvantaged economic situation, which can also affect health and wellbeing during childhood. For this reason, we calculated raw PD and PR and also adjusted by social class, as a proxy of the family’s socioeconomic position. Adjusted PD were calculated with the adjusted prevalence obtained from the same Poisson regression model through the marginal standardisation method [46]. In all the analyses we took into consideration the complex sample design. We used the R software (version 3.6.3, R Foundation for Statistical Computing, Viena, Austria).

## 3. Results

### 3.1. EP by Sociodemographic and Socioeconomic Characteristics and Housing Conditions

Table 1 describes the sociodemographic and socioeconomic characteristics and housing conditions of the sample, as well as the percentage of EP according to these characteristics and conditions. In Barcelona, 10.6% of children lived in EP. The most affected age group was 4–11 year-olds, in which the percentage of children living in EP was 13%. There was a clear gradient in the percentage of children living in EP according to the parents’ country of birth, being 6.6% in children with both parents born in HI countries, 18.2% in those with one parent born in an HI country and another in an LMI country and 20.7% in those with both parents born in a LMI country. Point estimates also suggested a gradient in the percentage of EP in relation to household composition. Single-parent families were worst affected, and especially if they were extended single-parent families.

The most significant differences in the percentage of EP were related to the socioeconomic situation. For example, the percentage of EP in households unable to afford an unforeseen expenditure of €750 with their own resources was 30.8%, compared with 2.3% in those that could. The percentage of EP in families with food insecurity was 53.4%. We also found differences according to tenure, where the EP percentage was 1% in paid-ownership households and 17.6% in market-rate renting households. Finally, there was a strong overlap between different dimensions of EP: 32.7% of households with arrears on utility bills in the last 12 months also lived in dwellings with inadequate temperatures.

### 3.2. EP and Children’s Health and Wellbeing

Table 2 shows the prevalence of health and wellbeing outcomes among children with and without EP, as well as the respective absolute (PD) and relative (PR) differences. Children living in EP had 7.70 (95%CI: 2.86, 20.72) times more poor health than those without EP and the respective PD was 12.2%. EP was also associated with poor mental health (PD: 11.6% and PR [95%CI]: 2.46 [1.21, 4.99]) and the most affected dimensions were peer (PD: 8.9% and PR [95%CI]: 2.48 [1.09, 5.63]) and emotional (PD: 8% and PR [95%CI]: 1.70 [0.86, 3.34]) problems. Children living in EP also had lower HRQoL, with an average of 2.57 points less in the Kidscreen. The prevalence of asthma (10.3% vs. 2.5%) and overweight (44.4% vs. 29.6%) was also higher in children living in EP, specifically, it was 4.19 (95%CI: 1.47, 11.90) and 1.50 (95%CI: 1.05, 2.15) times more frequent than in children without EP, respectively. After adjustment of the models by social class, the association between EP and poor health was consistent and, for the rest of the outcomes examined, children living in EP were still more likely to have poorer results, although the differences were not statistically significant (at the 5% level).

## 4. Discussion

### 4.1. Main Findings

This study shows a high percentage of EP in families with children in the city of Barcelona, as well as inequalities by sociodemographic and socioeconomic characteristics and housing conditions. We found a strong association between EP and poor health in children. The findings also suggest that EP is associated with poorer mental health, lower HRQoL and with more cases of asthma and overweight in children.

### 4.2. Interpretation of Results

In this study, 10.6% of children in Barcelona were living in EP. A value well above the EU average according to the most up-to-date data from the EU Energy Poverty Observatory, which indicate that in 2018, 7.3% of the EU population was not able to keep their home adequately warm. This result is consistent with the percentages found in a previous study in adults, reporting that 13.3% of women and 11.3% of men in the city of Barcelona lived in EP [7]. Robustly, both studies show how the axes of inequality that place our society in a hierarchy lead to greater exposure to EP in more vulnerable groups such as people born in LMI countries, those from more disadvantaged social classes and women. In this study, we found no differences between sexes. This may be because women are more affected by EP in adulthood due to gender roles that make them more responsible for reproductive and caregiving activities [47] and to the lesser economic, political and social power that women have in patriarchal societies, which hinders their access to commoditized goods such as energy. These aspects do not yet manifest at such early ages. However, gender differences can be observed related to household composition. As in other studies [18,24], in our study the prevalence of EP was higher in single-parent households, which are generally headed by women (in 85.4% of those in the study population).

We found slightly lower percentages of EP in households with children aged 0–3 years. This may be because infants and toddlers are more vulnerable to extreme variations in ambient temperatures due to their immature physiologic capacity for thermoregulation [21] and parents may choose strategies to cope with EP other than self-rationing of energy consumption. Not being able to keep the house at an adequate temperature is only one of the many expressions of EP. Families who decide to go into debt or reduce other basic needs to pay for energy services and those who are forced to connect irregularly to the distribution network also suffer from EP [11]. These situations often coexist in the same household, as found in this study where families with arrears on utility bills also had greater difficulty in keeping their homes at an adequate temperature. This can place a double burden on health, due to the specific direct effects of each dimension of EP.

Because the current energy model turns basic supplies into a commodity accessible only to those able to pay for them, EP usually occurs in disadvantaged socioeconomic contexts. For this reason and as also shown in this study, EP generally coexists with other economic and material hardship, such as job, food or housing insecurity [8,10,21,22,48]. This interaction of difficulties increasingly results in deteriorating health and makes it more difficult to break out of the circle of precariousness.

The most striking result in this study was the strong association between EP and poor child health. After adjustment for social class, children living in EP had 6.51 times more poor health than children without EP. Very few studies have quantified the magnitude of the association between EP and children’s health. A United States study that also examined the association between EP and poor health in children younger than 36 months found that children in households with moderate or severe EP had an adjusted odds of poor health 30% higher than those in households without EP. The authors suggest that there is a low “threshold effect” of EP on children’s health, affecting both moderate and severe cases of EP [21].

EP has been found to predominantly affect physical health in children and mental health in adolescents, but there have been few systematic assessments of the mental health effects on children [13]. In this study, we assessed the effects of EP on mental health in 435 young people, 319 children aged between 4 and 11 years and 116 young adolescents aged between 12 and 14 years. The results suggest worse mental health in young people living in EP. The most affected scales were emotional and peer problems, which are those composing the SDQ internalising score. Internalising behaviours include anxiety, withdrawal and dysphoria [49]. Although measured with another scale, a recent study found that 9-year-old children experiencing dual food and energy hardship had a greater odds of coexisting internalising and externalising behaviours [48]. Mental health in children living in EP may be affected by different mechanisms; for example, by the effects of EP on physical health, since children’s mental health is more adversely affected by illness than adults health [13]. Stress and other mental health problems experienced by parents living in EP can be transferred to children, and can also negatively affect parental relationships and parenting behaviour [50]. In addition, children with EP may find their play space and opportunities for leisure and socialisation reduced [24].

So far, asthma has been the most widely studied health effect of EP in children. Previous studies have shown that asthma is associated with cold homes and damp and mouldy conditions, and that interventions to reduce EP improve asthma symptoms in children and reduce school absence [19,51,52,53]. The present study found a much higher percentage of children with asthma in households with EP. Although this result was not statistically significant after adjustment for social class, point estimation revealed that, in more temperate climates, there is also a strong association between EP and asthma, with children with EP having almost 3 times more asthma than those without EP.

Finally, the relationship between EP and poor nutrition in children is also a major concern. Families with EP have greater difficulty in buying healthy, quality food and in preserving and cooking it properly. Poor nutrition in children can lead to underweight and overweight, with significant long-term consequences for their health and wellbeing. One study showed that infants up to the age of 3 living in EP were 29% more likely to be underweight, but the effects of EP on child overweight have not yet been studied. In this study we found a much greater prevalence of overweight in children with EP, suggesting that children living in EP were 50% more likely to be overweight.

### 4.3. Limitations and Strengths

The main limitation of this study is the sample size. The low statistical power did not allow us to show many statistically significant associations after adjustment of the models for social class, and the low number of observations limited us to stratifying the analyses by possible effect modifiers. Another limitation is that the survey was completed by caregivers. Children and adolescents have valid opinions that should be collected. The lived experience of children living in EP is essential to better understand and scale how EP affects their health and wellbeing and build meaningful policy and practice to address EP [53]. Moreover, there are successful experiences of studies of EP with the participation of adolescents [26]. It should also be noted that this study was conducted in the city of Barcelona and may not be generalizable to other contexts. Finally, data are from 2016. Although these are the latest data available, they may be outdated, especially due to the exceptional circumstances caused by the Covid-19 this past year. This study, however, is the first to analyse and quantify the magnitude of the effects of EP on various child health and wellbeing outcomes in southern Europe and its relevant findings should draw the attention of practitioners, researchers and policy-makers to further advance this much-needed field.

## 5. Conclusions and Recommendations

EP is an important public health problem in the city of Barcelona, with a highly uneven distribution among families and that negatively affects children’s health and wellbeing. The poorer physical and mental health experienced by children living in EP has an immediate effect on their quality of life, but may also have important long-term health implications and may hamper children’s learning and socialising. Long-term health problems and a lower educational achievement can, in turn, affect employment opportunities and economic wellbeing in adulthood, increasing health inequalities and perpetuating cycles of precariousness, which may even affect future generations.

These serious consequences in the short- and long-term should prompt policy-makers to develop specific measures that prioritise children and adolescents and guarantee basic supplies for their favourable development and wellbeing. This is especially important in the context of the COVID-19 pandemic, when we are on the verge of a possible new economic crisis, which could drag many families into EP and aggravate its effects on health.

## Figures and Tables

**Table 1:** Description of the sample and percentages of energy poverty by sociodemographic and socioeconomic characteristics and housing conditions. Barcelona 2016.

	Total		Energy Poverty *			
	*n*	%	Cases	%	95%CI	*p*-Value †
**Total**	481	100	48	10.6	(7.7, 13.4)	
**Sociodemographic characteristics**						
**Sex**						
Girl	240	48.7	21	9.2	(5.4, 12.9)	0.367
Boy	241	51.3	27	11.9	(7.5, 16.2)	
**Age (years)**						
0–3	89	17.9	5	6.3	(1.1, 11.4)	0.091
4–11	284	61.7	35	13.0	(8.8, 17.1)	
12–14	108	20.3	8	7.0	(2.3, 11.8)	
**Parents country of birth**						
Both HI	339	70.6	21	6.6	(3.7, 9.6)	**<0.001**
One HI, one LMI	38	7.6	6	18.2	(5.0, 31.4)	
Both LMI	103	21.7	21	20.7	(12.7, 28.7)	
**Household composition**						
Single-parent	43	8.6	5	11.4	(1.8, 20.9)	0.553
Extended single-parent	39	7.9	6	16.4	(4.3, 28.5)	
Two-parents	350	72.8	31	9.4	(6.2, 12.7)	
Extended two-parents	49	10.7	6	13.2	(3.1, 23.3)	
**Socioeconomic characteristics**						
**Social class**						
Non-manual laborer	341	71.2	13	4.5	(1.9, 7.1)	**<0.001**
Manual laborer	134	28.8	34	25.2	(17.7, 32.7)	
**Can afford unforeseen expenditure of 750€**						
Yes	336	70.7	6	2.3	(0.3, 4.4)	**<0.001**
No	137	29.3	42	30.8	(22.9, 38.6)	
Arrears on utility bills in the last 12 months **						
No	337	82.7	23	7.3	(4.2, 10.3)	**<0.001**
Yes	70	17.3	22	32.7	(21.5, 43.9)	
**Food insecurity**						
No	432	89.9	22	5.7	(3.3, 8.2)	**<0.001**
Yes	49	10.1	26	53.4	(39.1, 67.6)	
**Tenure status**						
Paid property	100	20.7	1	1.0	(−1.0, 3.0)	**<0.001**
Property paying mortgage	169	36.2	12	6.9	(3.0, 10.7)	
Rent at market price	184	39.1	30	17.6	(11.7, 23.5)	
Social renting	5	1.1	0	0.0	(0.0, 0.0)	
Other	13	2.9	5	40.7	(14.0, 67.5)	
**Housing conditions**						
**Leaks, dampness in walls, floors, ceilings or foundations, or rot in floors, window frames or doors**						
No	432	89.7	37	9.1	(6.2, 11.9)	**0.003**
Yes	49	10.3	11	23.5	(11.3, 35.7)	
**No means of heating or central heating or room-heating appliances, but not used when necessary**						
No	444	92.5	35	8.3	(5.6, 11.0)	**<0.001**
Yes	35	7.5	13	38.5	(22.1, 54.9)	
**No air conditioner or air conditioner, but not used when necessary**						
No	251	52.2	2	0.9	(−0.4, 2.1)	**<0.001**
Yes	228	47.8	46	21.2	(15.6, 26.7)	

* Three missing values; ** 74 missing values; *n* = sample size; cases: number of children with EP; 95%CI: 95% confidence interval; HI: high-income; LMI: low- and middle-income; †: Pearson chi-square test with the second-order Rao-Scott correction, differences are considered statistically significant if *p*-value is less than 0.05 (marked in bold).

**Table 2:** Health measures prevalence among children with and without energy poverty and crude and adjusted absolute (prevalence difference—PD) and relative (prevalence ratio—PR) differences. Barcelona 2016.

	Energy Poverty (*n* = 48)		No Energy Poverty (*n* = 430)		PD	PR (95%CI)	aPD	aPR (95%CI)
	Cases	% (95%CI)	Cases	% (95%CI)				
**Poor health**	7	14.0 (4.1, 23.9)	8	1.8 (0.6, 3.1)	12.2	**7.70 (2.86, 20.72)**	10.3	**6.51 (2.45, 17.29)**
**Mental health: SDQ for age 4 to 14**								
Poor mental health (Total difficulties score)	9	19.6 (7.7, 31.4)	28	8.0 (5.1, 10.8)	11.6	**2.46 (1.21, 4.99)**	6.5	1.78 (0.91, 3.51)
Conduct problems	9	19.7 (7.8, 31.6)	49	14.2 (10.5, 17.9)	5.5	1.39 (0.72, 2.69)	1.1	1.08 (0.56, 2.08)
Hyperactivity	7	16.6 (5.1, 28.0)	42	12.2 (8.7, 15.7)	4.4	1.36 (0.64, 2.88)	1.2	1.09 (0.53, 2.26)
Emotional problems	9	19.6 (7.8, 31.4)	40	11.5 (8.1, 15.0)	8.0	1.70 (0.86, 3.34)	3.6	1.30 (0.68, 2.51)
Peer problems	7	14.9 (4.4, 25.3)	21	6.0 (3.5, 8.5)	8.9	**2.48 (1.09, 5.63)**	5.6	1.89 (0.81, 4.40)
Health-related quality of life: Kidscreen for age 6 to 14 *		59.7 (54.5, 64.9)		62.3 (60.8, 63.7)		−2.57 (−8.03, 2.89)		−2.61 (−8.94, 3.72)
**Asthma**	5	10.3 (1.5, 19.2)	11	2.5 (1.0, 3.9)	7.9	**4.19 (1.47, 11.90)**	4.8	2.84 (0.91, 8.80)
**Overweight**	22	44.4 (29.9, 59.0)	127	29.6 (25.3, 34.0)	14.8	**1.50 (1.05, 2.15)**	6.4	1.21 (0.84, 1.74)

SDQ: Strengths and Difficulties Questionnaire; *n* = sample size; cases: number of children with the health outcome; 95%CI: 95% confidence interval; PD: crude Prevalence Difference; PR: crude Prevalence Ratio; aPD: social class-adjusted Prevalence Difference; aPR: social class-adjuste Prevalence Ratio; * Kidscreen is the only continuous variable and therefore the values shown are means and their 95%CI among children with and without EP and mean differences and their 95%CI calculated through linear models; in bold statistically significant PR.

## Data Availability

Data available upon request.
